# How Immunocompromised Hosts Were Left Behind in the Quest to Control the COVID-19 Pandemic

**DOI:** 10.1093/cid/ciae308

**Published:** 2024-06-03

**Authors:** Michael Boeckh, Steven A Pergam, Ajit P Limaye, Janet Englund, Lawrence Corey, Joshua A Hill

**Affiliations:** Vaccine and Infectious Disease Division, Fred Hutchinson Cancer Center, Seattle, Washington, USA; Department of Medicine, Division of Allergy and Infectious Diseases, University of Washington, Seattle, Washington, USA; Vaccine and Infectious Disease Division, Fred Hutchinson Cancer Center, Seattle, Washington, USA; Department of Medicine, Division of Allergy and Infectious Diseases, University of Washington, Seattle, Washington, USA; Department of Medicine, Division of Allergy and Infectious Diseases, University of Washington, Seattle, Washington, USA; Department of Medicine, Division of Allergy and Infectious Diseases, University of Washington, Seattle, Washington, USA; Seattle Children's Research Institute, Seattle, Washington, USA; Vaccine and Infectious Disease Division, Fred Hutchinson Cancer Center, Seattle, Washington, USA; Department of Medicine, Division of Allergy and Infectious Diseases, University of Washington, Seattle, Washington, USA; Vaccine and Infectious Disease Division, Fred Hutchinson Cancer Center, Seattle, Washington, USA; Department of Medicine, Division of Allergy and Infectious Diseases, University of Washington, Seattle, Washington, USA

## Abstract

The immunocompromised population was disproportionately affected by the severe acute respiratory syndrome coronavirus 2 pandemic. However, these individuals were largely excluded from clinical trials of vaccines, monoclonal antibodies, and small molecule antivirals. Although the community of scientists, clinical researchers, and funding agencies have proven that these therapeutics can be made and tested in record time, extending this progress to vulnerable and medically complex individuals from the start has been a missed opportunity. Here, we advocate that it is paramount to plan for future pandemics by investing in specific clinical trial infrastructure for the immunocompromised population to be prepared when the need arises.

## Teaser

Early prevention and treatment trials for severe acute respiratory syndrome coronavirus 2 largely excluded immunocompromised individuals, neglecting vulnerable population. Regulatory requirement for trials and establishment of a clinical trial infrastructure focused on this population can address this gap in public health and advance pandemic preparedness.

## IMMUNOCOMPROMISED HOSTS WERE LAST IN LINE

Early in the coronavirus disease 2019 (COVID-19) pandemic, immunocompromised hosts were found to be severely affected by severe acute respiratory syndrome coronavirus 2 (SARS-CoV-2). In particular, transplant recipients and persons with hematologic malignancies carried a disproportionate burden of disease, with significant excess morbidity and mortality [1, 2].

Immunocompromised persons with a diverse array of immunodeficiencies, including untreated or incompletely controlled human immunodeficiency virus (HIV), exhibited prolonged shedding of the virus because of their inadequate eradicative immune response [3, 4]. This inability to eliminate infection favored viral evolution and has been a theorized source for the development of new variants [5]. Genetic and epidemiologic analyses of the origins of the Alpha, Beta, and Omicron variants of concern indicate their likely origin in an immunocompromised host, illustrating the importance of developing and prioritizing strategies to prevent persistent SARS-CoV-2 infection and develop eradication strategies in such persons [5, 6].

Estimates indicate 3%–6% of the US and European population are affected by immunosuppressive conditions [7, 8]. For countries with a high prevalence of untreated and undiagnosed HIV, this figure may even higher. The spectrum of immunosuppressive conditions beyond transplantation and hematologic and nonhematologic malignancies that may increase risk for SARS-CoV-2 infection and disease severity is broad. An expanding proportion of the population at risk includes those with autoimmune conditions, genetic and chromosomal conditions with inherent host defense alterations [9], recipients of novel cellular and monoclonal antibody therapies [10, 11], and untreated or poorly controlled HIV [12, 13].

**Figure 1.**
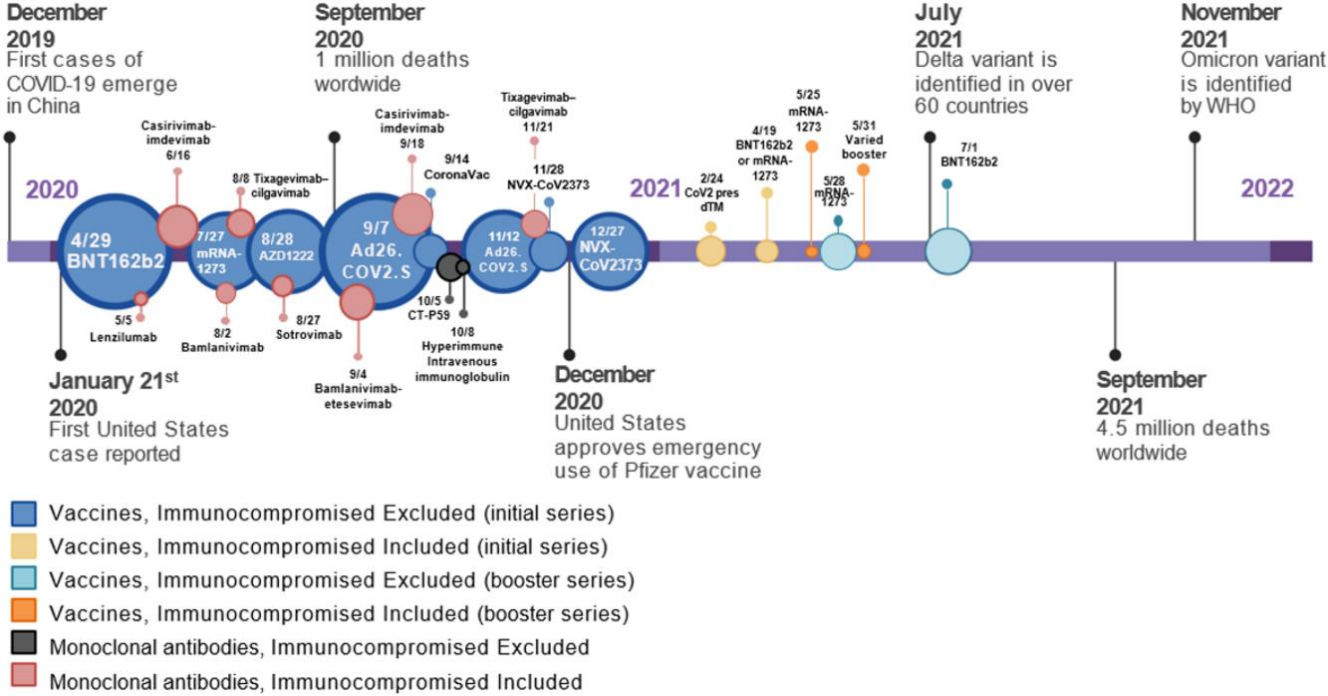
Timing of key vaccine trials and monoclonal antibody trials during the early phase of the SARS-CoV-2 pandemic (trial details are located in Supplementary Table 1) and the inclusion status of immunocompromised participants. The color of the circles indicates participation of immunocompromised individuals in vaccine trials (dark blue: initial series/excluded; yellow: initial series/included; light blue: booster series/excluded; orange: booster series/included) or monoclonal antibody and immunoglobulin product trials (gray: immunocompromised excluded, red: immunocompromised included), whereas the size shows the relative size of the trials. Abbreviations: COVID-19, coronavirus disease 2019; SARS-CoV-2, severe acute respiratory syndrome coronavirus 2.

Virtually all initial trials evaluating novel vaccines, monoclonal antibodies (mAbs), and antiviral drugs for SARS-CoV-2 excluded immunosuppressed individuals from participation (Figure 1). If not excluded, this population accounted for a small fraction of enrolled participants, precluding meaningful subgroup analysis. The rationale for this decision in the vaccine program was historically poor response rates of vaccines in immunocompromised patients [14, 15], theoretical concerns for vaccine-enhanced disease complications (eg, organ rejection) [16, 17], and worry that expected higher baseline rate of severe adverse events in clinical trials in immunocompromised patients might impact efficacy or even licensure issues [18], among others (Table 1). For mAbs and antiviral drugs in this population, concerns also included altered pharmacokinetics, especially with the wide range of medications taken by immunocompromised patients, uncertainty about doses or if regimens required for mAbs and antivirals might differ substantially from immunocompetent persons, and theoretical complications (eg, organ rejection), among others. These concerns, coupled with the need for rapid clinical trial accrual to address an ongoing pandemic and the uncertain infection rate for predicting sample size, led to the exclusion of all but the most mildly immunocompromised persons into the pivotal trials of vaccines, mAbs, and antivirals in both government and industry sponsored trials, not only in 2020 but up to the present time.

**Table 1.** Consideration for Early Inclusion of Immunocompromised Individuals Into Clinical Trials of Novel Vaccines, Monoclonal Antibodies, and Small Molecules

Issue	Potential Significance	Evidence in Immunocompromised Populations	Mitigation
**Vaccine Trials**			
Reduced immunogenicity	Adds variability to trial results that may require stratification of enrollment and associated statistical implications (sample size) May affect overall response rates	Strong evidence but less significant with more recent vaccines and in mildly or moderately immunosuppressed individuals Discordance between antibody and cellular responses	Separate parallel trials in immunocompromised hosts Incorporate additional boosters or consider higher doses Broad assessment of immunogenicity
High background rate of AEs and SAEs	Well-established from prior trials May bias interpretation if immunocompromised hosts are coenrolled with healthy participants	Strong evidence	Separate parallel trials DSMB oversight Proactive communication to the public
Vaccine-enhanced disease	Increased disease severity when infected with wild-type pathogen (first-generation RSV vaccines, Dengue vaccine) Rare but concerning phenomenon with novel, first in humans vaccines	No evidence to date in immunocompromised populations Effect likely less important than in the general population because of immunosuppression	Enhanced trial oversight by DSMB
Vaccine-related “off-target” immune reactions	Induction of GVHD after allogeneic HCT Increased acute or chronic allograft rejection in SOT recipients Flare of disease activity in immune-mediated diseases (eg, multiple sclerosis)	To date, no clear evidence of GVHD linked to vaccines after allogeneic HCT or acute or chronic allograft rejection in SOT recipients Multiple sclerosis—evidence weak	Conduct initial trials in lower risk settings (eg, autologous HCT, kidney transplantation) Enhanced trial oversight by DSMB
**Monoclonal antibody and immunoglobulin product trials**			
High background rate of AEs and SAEs	Maybe taken out of context if part of trials in otherwise healthy participants	Well-known phenomenon Strong evidence	Separate trials DSMB oversight Careful explanations when communicating trial results
Loss of target	Diminished or lost efficacy because of emergence of variants, antigenic shift	Strong evidence	Higher doses in selected circumstances Development of broadly neutralizing antibodies, increased resistance to viral evasion
Different pharmacokinetics	May affect dosing intervals because of increased clearance	Moderate-strong for gastrointestinal GVHD after allogeneic HCT	Incorporate in study design May require shorter dosing intervals and/or higher dosing
Immune enhancement phenomenon	Theoretical concern, increased disease severity	Limited evidence to date	Enhanced trial oversight by DSMB
**Small molecule trials**			
High background rate of AEs and SAEs	Well-known phenomenon Maybe taken out of context if part of trials in otherwise healthy participants	Strong evidence	Separate trials DSMB oversight Careful explanations when communicating trial results
Drug–drug interactions	Increased toxicities, altered immunosuppression	Strong, eg, nirmatrelvir/ritonavir (COVID-19)	Adjust entry criteria Temporarily hold drugs if possible Therapeutic drug monitoring as needed
Different pathogen kinetics	Insufficient treatment effect Emergence of resistance	Strong evidence for prolonged shedding Propensity for emergence of resistance and variants depends on both the pathogen and specific drug pressure	Incorporate different dosing and durations into the study design (eg, efficacy endpoints) Intensify pathogen and resistance surveillance
Altered pharmacokinetic or pharmacodynamics	Higher doses and prolonged treatment courses may be needed Different toxicities may occur because of exposure and preexisting conditions	Strong evidence	Separate studies of different doses and durations
Emergence of resistance	Diminished or loss of efficacy	Limited evidence of clinical significance	Higher doses Combination therapy

Abbreviations: AE, adverse event; COVID-19, coronavirus disease 2019; DSMB, data safety monitoring board; GVHD, graft versus host disease; HCT, hematopoietic cell transplantation; RCT, randomized controlled trial; RSV, respiratory syncytical virus; SAE, serious adverse event; SOT, solid organ transplantation.

One potential solution to this problem would have been the development and execution of parallel trials in immunocompromised hosts to identify efficacious dosing strategies and/or regimens for vaccines, mAbs, and antivirals. This parallel track approach was not undertaken and only very recently, several years after the start of the epidemic, results of randomized trials large enough to establish safety and efficacy of SARS-CoV-2 vaccines, mAbs, or small molecules in immunocompromised hosts are available in the literature. To fill the gap, and driven by high demand and desperation, the community of physicians who care for immunocompromised patients created guidelines to use the novel mRNA vaccines once available—initially in a data vacuum, and later based on accumulating reports in the literature of mostly small, single-center retrospective case series [19, 20]. As the pandemic progressed and ultimately ended, meta-analyses of immunogenicity from available data were another avenue attempted to bridge the gap for immunocompromised patients; however, this represents another delayed response for this vulnerable population [15, 21]. Here, we summarize the lack of inclusion of immunocompromised hosts in assessing the initial immunotherapeutic and pharmacologic response of new therapies for SARS-CoV-2, outline consequences of this approach, and propose solutions to address the problem in the future.

## LACK OF INCLUSION OF IMMUNOCOMPROMISED HOSTS IN CLINICAL TRIALS OF VACCINES, MONOCLONAL ANTIBODIES, AND SMALL MOLECULE ANTIVIRALS

A review of phase 3 clinical trials of SARS-CoV-2 vaccine and mAb trials indicates the uniform exclusion of moderately and severely immunocompromised participants during the first year of development with 1 exception (Supplementary Table 1) [22]. A timeline of the trials in different populations is shown in Figure 1. By November 2020, data emerged of the high efficacy of the 2-dose 100-mcg Moderna or 30-mcg BioNTech-Pfizer regimens, and emergency use authorizations (EUAs) for adult immunization of the US populace were released in December 2020, with vaccination rollout in January 2021. One of the confounding issues about the issuance of an EUA status was that when these products were considered investigational, off-label uses such as higher and/or multiple doses were not allowed.

The community of clinicians involved in the care of immunocompromised persons, including people with HIV, began investigator-initiated safety and immunogenicity protocols in a wide variety of immunocompromised populations with limited funding and without intent for regulatory submission. Common features of these reports were small sample size from single centers, nonstandardized immunogenicity, nonstandardized laboratory endpoint assessments, and retrospective designs, resulting in an overall low-quality evidence base. It was not until later in 2021 that a few investigator-initiated trials and prospective cohort studies specifically designed for immunocompromised populations were conducted, primarily to evaluate booster strategies without substantial funding from government sources (eg, National Institutes of Health [NIH]) in most instances (Supplementary Table 1, Figure 1), although some investigator-initiated studies received NIH funding. Ultimately, EUAs were amended by the Food and Drug Administration on 12 August 2021 to allow for additional vaccine doses in immunocompromised hosts [23]. However, no clinical trials were conducted to enable analyses of correlates of protection of vaccine efficacy, and studies were not conducted in immunocompromised children. Studies in immunocompromised persons were neither designed nor funded to use the laboratory facilities used by the Operation Warp Speed program in the United States, which would have allowed direct comparison with the gold standard recommendations made by the Food and Drug Administration for immune competent persons.

The evaluation of mAbs for both treatment and prophylaxis in immunocompromised individuals followed a somewhat different path as it was recognized early that mAbs could be of particular value for immunocompromised persons who were expected to respond poorly to active immunization [24]. Dosing, relative efficacy, concerns for antibody-mediated enhancement, and the rapid development of resistance by immunocompromised persons were all initial concerns for exclusion of immunocompromised patients. Once efficacy was observed in immunocompetent trial participants, several studies started including immunocompromised patients [25, 26] (Figure 1), but they accounted for only a small proportion of trial participants. Subsequently, larger studies were planned, but the development of variants rendered available products ineffective [27]. Of note, parallel track studies specifically focused on highest risk immunocompromised populations were not performed in the early phase of the pandemic [24].

The development of small molecule inhibitors such as remdesivir, molnupiravir, and nirmatrelvir/ritonavir followed a similar paradigm as for other interventions [28, 29]. Although immunocompromised participants were not explicitly excluded from most small molecule trials, as in the case of the vaccine trials, the immunocompromised status of patients was not thoroughly analyzed or described in the major publications, resulting in a similar lack of data applicable to the immunocompromised population (see remdesivir supplemental literature). Trials that attempted to address the data gaps in immunocompromised hosts, including viral load suppression, rebound infection, development of resistance, and dose and duration of antiviral treatment were initiated late, enrolled slowly, and at least 1 was discontinued [30–32]. Moreover, no compassionate use protocols for extended use antiviral drugs were available to immunocompromised patients, especially with severe lower respiratory infections. In fact, the EUA for nirmatrelvir/ritonavir, the antiviral with the best clinical potency, did not allow administration to hospitalized patients. At the behest of many advocates for the immunocompromised patients, a study of prolonged administration of nirmatrelvir/ritonavir in hospitalized immunocompromised patients was eventually initiated but results are not available yet. Evaluation of similar versus alternative dosing and duration strategies, as well as combination therapies, is particularly important in immunocompromised patients with limited immunologic function to contribute to viral control and clearance but was largely overlooked.

## PANDEMIC CONTROL MUST INCLUDE THE EARLY DEVELOPMENT OF VACCINES AND THERAPEUTICS SPECIFICALLY FOR THE MOST VULNERABLE POPULATIONS

The greatest omission was the lack of coordinated funding from the US government, including the NIH, for initiating the multicenter organizational structure to establish the evidence for efficacy of preventive and treatment strategies for COVID-19 in immunocompromised patients. Although individual institutes of the NIH sponsored some safety and immunogenicity studies in patient groups in their purview populations, the resultant studies were neither powered nor funded to provide estimates of vaccine or monoclonal antibody efficacy and were not linked to the laboratory and statistical infrastructure established for immunocompetent persons to allow any relative comparison to the immune response or linking to the correlates of protection program associated with these large-scale studies.

There were clearly perceived barriers in executing trials in immunocompromised individuals during the early phase of the pandemic. However, as we outline in Table 1, we believe these are surmountable, especially for vaccines and mAbs, in which there are few direct drug interactions with typical medications for immunocompromised patients. To address safety concerns as well as possible off-target effects, an enhanced oversight of these studies could be employed through designation of adverse events of special interest and more frequent data safety monitoring board (DSMB) review. The recent positive results with novel vaccines in transplant recipients against cytomegalovirus [33] and varicella zoster virus [34], which already led to expanded approval in immunocompromised patients for a previously licensed vaccine to prevent herpes zoster [35], provide examples for the feasibility of successful vaccine development in immunocompromised populations. Overall, the lack of a regulatory mandate to conduct and fund such trials is an important reason why trials were not conducted during the pandemic. Immunocompromised children are perceived to present additional complexities, such as differences in pharmacokinetics and immunogenicity, age-specific adverse effects, and different risk-benefit considerations (eg, pericarditis with mRNA vaccines). However, solutions to these appear to be similarly manageable (Table 1) and do not seem to justify an a priori exclusion from clinical trials. Indeed, profoundly immunosuppressed children may be at similarly high risk of pathogen-related complications, balancing or even outweighing specific concerns related to clinical trial participation.

Future studies of small molecules evaluating specific questions in immunocompromised patients should also be initiated concurrently with trials in the immunocompetent individuals, potentially using viral load as the primary endpoint [36, 37], and including the evaluation of drug combinations from the beginning [38, 39].

## CONCLUSION AND FUTURE NEEDS

The immunocompromised population was disproportionately affected by the SARS-CoV-2 pandemic to a large degree because of the absence of the development of effective preventive and therapeutic strategies to these most vulnerable persons during the early phase of the pandemic. This omission led to considerable measured and unmeasured morbidity in affected people [1]. Although the community of scientists, clinical researchers, and funding agencies have proven that vaccines, mAbs, and small molecules can be made and tested in record time, extending this progress to vulnerable and medically complex individuals from the start has been a major missed opportunity. This has occurred despite the expertise of scientists in designing protocols for randomized placebo-controlled vaccine trials in these patients.

**Figure 2.**
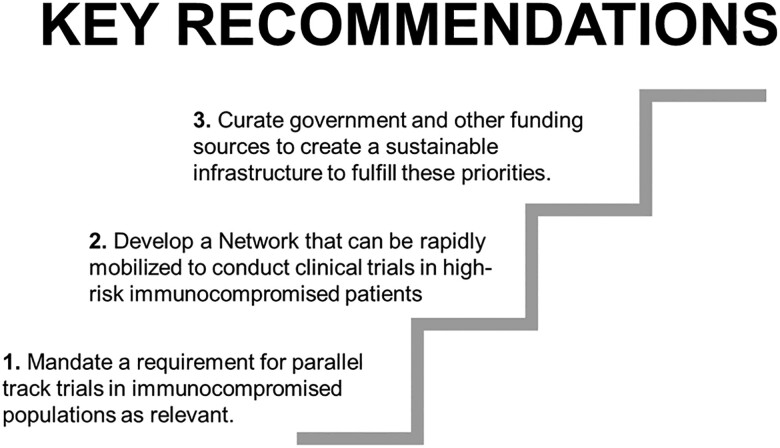
Key recommendations for developing robust infrastructure for immunocompromised inclusive clinical trials.

The enormity of the task to rapidly initiate SARS-CoV-2 vaccine, monoclonal, and drug therapy programs for the global population was overwhelming and fell short in including what now seems like a largely forgotten patient population and investigative community. Thus, it is paramount to plan for future pandemics by investing in specific clinical trial infrastructures for the immunocompromised population and to be prepared when the need arises (Figure 2). To accomplish this, dedicated funding from government sources, such as the NIH, will be required. Given the scope of the task, pooled funding from various institutes within the NIH may be necessary. Equally important is to enact a regulatory mandate to conduct parallel clinical trials in immunosuppressed patients, including children, early in the development process. Concurrent investments are needed to fund research aimed at understanding the impact of immunologic deficits on infection risk and vaccine response across the spectrum of immunosuppression [40], as well as developing specific strategies for overcoming these deficits (eg, dose and frequency of vaccination, timing of vaccination, adjuvants).

The proposed actions need to be implemented now to prevent a similar failure to act when the next pandemic strikes.

## Supplementary Data

Supplementary materials are available at *Clinical Infectious Diseases* online. Consisting of data provided by the authors to benefit the reader, the posted materials are not copyedited and are the sole responsibility of the authors, so questions or comments should be addressed to the corresponding author.

## Supplementary Material

ciae308_Supplementary_Data

## Notes

***Acknowledgments.*** The authors thank Alex Downing for assistance in manuscript preparation.
