# Supplementary material for: How Immunocompromised Hosts Were Left Behind in the Quest to Control the COVID-19 Pandemic
Source: Clin Infect Dis. 2024 Jun 3;79(4):1018–23. doi: 10.1093/cid/ciae308 (PMC11478583; doi:10.1093/cid/ciae308)
Supplement: ciae308_Supplementary_Data [file ciae308_supplementary_data.docx]

**Supplementary Material**

**Supplementary Table 1**. Inclusion of immunocompromised participants in major randomized controlled SARS-CoV-2 vaccine and monoclonal antibody trials during the early phase of the SARS-CoV-2 pandemic (trials start days between 4-2020 and first half of 2021).

| **Compound** | **Manufacturer/ Testing Partner** | **Year** | **Trial Phase** | **Topic** | **Participant Age** | | | | **Inclusion Criteria** | | **Reference Publication** |  |
| --- | --- | --- | --- | --- | --- | --- | --- | --- | --- | --- | --- | --- |
|  |  |  |  |  |  |  |  |  | **Immunosuppression** | |  |  |
|  |  |  |  |  | **median (range)** | **median (IQR)** | **mean (range)** | **mean (IQR)** | **Moderate ^C^** | **Severe ^D^** |  |  |
| **Vaccine Trials** | | | | | | | | | | | |  |
| BNT162b2 | Pfizer | 2020 | 2\|3 | Initial Series | 51  (16-91) |  |  |  |  |  | Thomas, et al. |  |
| ChAdOx1 nCoV-19 (AZD1222) | University of Oxford | 2020 | 2\|3 | Initial Series | (22-76) |  |  |  |  |  | Ramasamy, et al. |  |
| mRNA-1273 | Moderna | 2020 | 3 | Initial Series | 51.4 (18-95) |  |  |  |  |  | Baden, et al. |  |
| AZD1222 (ChAdOx1 nCoV-19) | AstraZeneca | 2020 | 3 | Initial Series | 51  (18-100) |  |  |  |  |  | Falsey, et al. |  |
| Ad26.COV2.S | Janssen Vaccines | 2020 | 3 | Initial Series | 52  (18-100) |  |  |  |  |  | Sadoff, et al. |  |
| CoronaVac | Sinovac | 2020 | 3 | Initial Series | 45  (37-51) |  |  |  |  |  | Tanriover, et al. |  |
| Ad5-nCoV | CanSino Biologics Inc. | 2020 | 3 | Initial Series |  |  |  | 37  (23-51) |  |  | Halperin, et al. |  |
| Ad26.COV2.S | Janssen Vaccines | 2020 | 3 | Initial Series |  | 53  (42-62) |  |  |  |  | Hardt, et al. |  |
| CoVLP+AS03 | Medicago | 2020 | 2\|3 | Initial Series | 32.8 (18-86) |  |  |  |  |  | Hager, et al. |  |
| NVX-CoV2373 | Novavax | 2020 | 3 | Initial Series | 55  (18-84) |  |  |  |  |  | Toback, et al. |  |
| CVnCoV | CureVac | 2020 | 2\|3 | Initial Series |  | 43  (31-54) |  |  |  |  | Kremsner, et al. |  |
| ZF2001 | Anhui Zhifei Longcom Biologic Pharmacy Co., Ltd. | 2020 | 3 | Initial Series | 35  (17-92) |  |  |  |  |  | Dai, et al. |  |
| QazCovid-in(R) | Research Institute for Biological Safety Problems\|City polyclinic No. 4 of the UZO of Almaty\|Clinic of the International Institute of Postgraduate Education\|City Multidisciplinary Hospital of the Health Department of the Akimat of Zhambyl Region | 2020 | 3 | Initial Series |  | 35  (26-46) |  |  |  |  | Khairullin, et al. |  |
| NVX-CoV2373 | Novavax | 2020 | 3 | Initial Series | 47  (18-95) |  |  |  |  |  | Dunkle, et al. |  |
| mRNA-1273 | Moderna | 2020 | 2\|3 | Initial Series |  | 14.3 (12.7-15.9) |  |  |  |  | Ali, et al. |  |
| BNT162b2 | Pfizer | 2021 | 2\|3 | Initial Series | 8  (5-11) |  |  |  |  |  | Walter, et al. |  |
| BBV152 | Bharat Biotech International Limited | 2021 | 2\|3 | Initial Series | 11  (2-18) |  |  |  |  |  | Mohan Vadrevu, et al. |  |
| CoV2 preS dTM | Sanofi Pasteur | 2021 | 2\|3 | Initial Series |  |  |  | 53  (38-68) |  |  | Sridhar, et al. |  |
| SCB-2019 | Clover Biopharmaceuticals AUS Pty Ltd\|Coalition for Epidemic Preparedness Innovations\|International Vaccine Institute | 2021 | 2\|3 | Initial Series |  |  | 32.1 (18-86) |  |  |  | Bravo, et al. |  |
| mRNA-1273 or BNT162b2 | University Hospital, Basel, Switzerland\|Swiss National Science Foundation\|ModernaTX, Inc. | 2021 | 3 | Initial Series |  | 53  (43-61) |  |  |  |  | Speich, et al. |  |
| VLA2001 or ChAdOx1-S | Valneva Austria GmbH | 2021 | 3 | Initial Series |  |  | 33  (18-71) |  |  |  | Lazarus, et al. |  |
| SpikoGen | Cinnagen\|Vaxine Pty Ltd | 2021 | 3 | Initial Series |  |  |  | 33  (26-40) |  |  | Tabarsi, et al. |  |
| mRNA-1273 | Moderna | 2021 | 2\|3 | Booster Series |  |  | 57  (20-96) |  |  |  | Chalkias, et al. |  |
| BNT162b2 | Pfizer | 2021 | 3 | Booster Series | 53  (16-87) |  |  |  |  |  | Moreira, et al. |  |
| SpikoGen | Cinnagen\|Vaxine Pty Ltd | 2021 | 3 | Booster Series |  |  |  | 45  (27-63) |  |  | Tabarsi, et al. |  |
| mRNA-1273 | Moderna | 2021 | 4 | Booster Series |  | 66.6 (63.3-71.4) |  |  |  |  | Hall, et al. |  |
| mRNA-1273 | Moderna | 2021 | 4 | Booster Series |  | 66.7 (63.6-71.4) |  |  |  |  | Kumar, et al.^A,^ |  |
| BNT16262 or mRNA-1273 or Ad26COVS1 | Medical University of Vienna | 2021 | 2 | Booster Series |  |  |  | 61.2 (12.4)^B^ |  |  | Reindl-Schwaighofer, et al. |  |
| **Monoclonal Antibody and Immunoglobulin Products Trials** | | | | | | | | | | | |  |
| Lenzilumab | Humanigen, Inc. | 2020 | 3 | Antibody Treatment |  |  | 62  (22-98) |  |  |  | Temesgen, et al. |  |
| Tixagevimab–cilgavimab | University of Minnesota\|International Network for Strategic Initiatives in Global HIV Trials (INSIGHT)\|University of Copenhagen\|Medical Research Council\|Kirby Institute\|Washington D.C. Veterans Affairs Medical Center\|AIDS Clinical Trials Group\|National Heart, Lung, and Blood Institute (NHLBI)\|US Department of Veterans Affairs\|Prevention and Early Treatment of Acute Lung Injury (PETAL)\|Cardiothoracic Surgical Trials Network (CTSN)\|Eli Lilly and Company\|Vir Biotechnology, Inc.\|GlaxoSmithKline\|Brii Biosciences Limited\|AstraZeneca\|Molecular Partners AG\|National Institute of Allergy and Infectious Diseases (NIAID)\|Pfizer | 2020 | 3 | Antibody Treatment |  |  | 55  (44-66) |  |  |  | Ginde, et al. |  |
| Sotrovimab | Vir Biotechnology, Inc.\|GlaxoSmithKline | 2020 | 2\|3 | Antibody Treatment | 53  (18-96) |  |  |  |  |  | Gupta, et al. |  |
| CT-P59 (Regdanvimab) | Celltrion | 2020 | 2\|3 | Antibody Treatment |  |  |  | 48  (38-59) |  |  | Streinu-Cercel, et al. |  |
| Hyperimmune intravenous immunoglobulin (hIVIG) | University of Minnesota\|National Institute of Allergy and Infectious Diseases (NIAID)\|National Institutes of Health (NIH)\|International Network for Strategic Initiatives in Global HIV Trials (INSIGHT) | 2020 | 3 | Antibody Treatment | 59  (49-70) |  |  |  |  |  | Polizzotto, et al. |  |
| Bamlanivimab | Eli Lilly and Company | 2020 | 3 | Antibody Treatment |  |  | 53 (18-104) |  |  |  | Cohen, et al. |  |
| Casirivimab-imdevimab | Regeneron Pharmaceuticals | 2020 | 3 | Antibody Treatment | 45 (0-96) |  |  |  |  |  | Weinreich, et al |  |
| Tixagevimab-cilgavimab | AstraZeneca | 2020 | 3 | Antibody Treatment |  |  |  | 53.52 (14.97)^B^ |  |  | Levin, et al |  |
| Bamlanivimab-etesevimab | Eli Lilly and Company | 2020 | 3 | Antibody Treatment |  |  |  | 51.9 (16.95) ^B^ |  |  | Dougan, et al |  |
| Casirivimab-imdevimab | University of Oxford | 2020 | 3 | Antibody Treatment |  |  |  | 61.9 (14.6) ^B^ |  |  | RECOVERY |  |
|  |  |  |  |  |  |  |  |  |  |  |  |  |
| Footnotes |  |  |  |  |  |  |  |  |  |  |  |  |

Blue boxes indicate that participants with immunocompromising conditions were allowed; Pink boxes indicate that participants with immunocompromising conditions were excluded. References are included in the supplementary material.

^A^ Secondary analysis of Hall, et al.

^B^ Standard deviation of the age is given

^C^ Moderate immunosuppression: solid tumor chemotherapy, cancer survivor, rheumatoid arthritis, multiple sclerosis, Down Syndrome

^D^ Severe immunosuppression: transplantation, hematologic malignancy chemotherapy, uncontrolled HIV infection, primary immunodeficiency

**Additional Supportive Literature**

General Literature

1. Shields AM, Burns SO, Savic S, Richter AG. COVID-19 in patients with primary and secondary immunodeficiency: The United Kingdom experience. The Journal of allergy and clinical immunology 2021; 147(3): 870-5.e1.
2. Bahremand T, Yao JA, Mill C, Piszczek J, Grant JM, Smolina K. COVID-19 hospitalisations in immunocompromised individuals in the Omicron era: a population-based observational study using surveillance data in British Columbia, Canada. Lancet Reg Health Am 2023; 20: 100461.
3. Belsky JA, Tullius BP, Lamb MG, Sayegh R, Stanek JR, Auletta JJ. COVID-19 in immunocompromised patients: A systematic review of cancer, hematopoietic cell and solid organ transplant patients. J Infect 2021; 82(3): 329-38.
4. Finckh A, Ciurea A, Raptis CE, Rubbert-Roth A. Susceptibility to COVID-19 and Immunologic Response to Vaccination in Patients With Immune-Mediated Inflammatory Diseases. The Journal of Infectious Diseases 2023; 228(Supplement_1): S13-S23.
5. Subramanian V. Susceptibility to SARS-CoV-2 Infection and Immune Responses to COVID-19 Vaccination Among Recipients of Solid Organ Transplants. J Infect Dis 2023; 228(Suppl 1): S34-s45.Mushtaq MU, Shahzad M, Chaudhary SG, et al. Impact of SARS-CoV-2 in Hematopoietic Stem Cell Transplantation and Chimeric Antigen Receptor T Cell Therapy Recipients. Transplantation and Cellular Therapy 2021; 27(9): 796.e1-.e7.
6. Mushtaq MU, Shahzad M, Chaudhary SG, et al. Impact of SARS-CoV-2 in Hematopoietic Stem Cell Transplantation and Chimeric Antigen Receptor T Cell Therapy Recipients. Transplantation and Cellular Therapy 2021; 27(9): 796.e1-.e7.
7. Descotes J. Immunotoxicity of monoclonal antibodies. MAbs 2009; 1(2): 104-11.
8. Caldera F, Mercer M, Samson SI, Pitt JM, Hayney MS. Influenza vaccination in immunocompromised populations: Strategies to improve immunogenicity. Vaccine 2021; 39 Suppl 1: A15-a23.
9. Hegazy AN, Krönke J, Angermair S, et al. Anti-SARS-CoV2 antibody-mediated cytokine release syndrome in a patient with acute promyelocytic leukemia. BMC Infectious Diseases 2022; 22(1): 537.
10. Bartoletti M, Azap O, Barac A, et al. European society of clinical microbiology and infectious diseases guidelines for coronavirus disease 2019: an update on treatment of patients with mild/moderate disease. Clin Microbiol Infect 2022; 28(12): 1578-90.
11. Cornberg M, Buti M, Eberhardt CS, Grossi PA, Shouval D. EASL position paper on the use of COVID-19 vaccines in patients with chronic liver diseases, hepatobiliary cancer and liver transplant recipients. J Hepatol 2021; 74(4): 944-51.
12. Elias KM, Khan SR, Stadler E, et al. Viral clearance as a surrogate of clinical efficacy for COVID-19 therapies in outpatients: A systematic review and meta-analysis. medRxiv 2023: 2023.06.18.23291566.
13. Chen P, Bergman P, Blennow O, et al. Real-world assessment of immunogenicity in immunocompromised individuals following SARS-CoV-2 mRNA vaccination: a one-year follow-up of the prospective clinical trial COVAXID. EBioMedicine **2023**; 94: 104700.
14. Hill JA, Martens MJ, Young JH, et al. SARS-CoV-2 vaccination in the first year after allogeneic hematopoietic cell transplant: a prospective, multicentre, observational study. EClinicalMedicine **2023**; 59: 101983.
15. Westblade LF, Brar G, Pinheiro LC, et al. SARS-CoV-2 Viral Load Predicts Mortality in Patients with and without Cancer Who Are Hospitalized with COVID-19. Cancer Cell **2020**; 38(5): 661-71.e2.
16. Bucciol G, Tangye SG, Meyts I. Coronavirus disease 2019 in patients with inborn errors of immunity: lessons learned. Curr Opin Pediatr **2021**; 33(6): 648-56.
17. Aldoss I, La Rosa C, Baden LR, et al. Poxvirus Vectored Cytomegalovirus Vaccine to Prevent Cytomegalovirus Viremia in Transplant Recipients: A Phase 2, Randomized Clinical Trial. Ann Intern Med **2020**; 172(5): 306-16.

Remdesivir Literature

1. Beigel JH, Tomashek KM, Dodd LE, et al. Remdesivir for the Treatment of Covid-19 — Final Report. New England Journal of Medicine **2020**; 383(19): 1813-26.

2. Goldman JD, Lye DCB, Hui DS, et al. Remdesivir for 5 or 10 Days in Patients with Severe Covid-19. New England Journal of Medicine **2020**; 383(19): 1827-37.

3. Spinner CD, Gottlieb RL, Criner GJ, et al. Effect of Remdesivir vs Standard Care on Clinical Status at 11 Days in Patients With Moderate COVID-19: A Randomized Clinical Trial. JAMA **2020**; 324(11): 1048-57.

**References for Supplementary Table 1. Inclusion of immunocompromised participants in major randomized controlled SARS-CoV-2 vaccine and monoclonal antibody trials during the early phase of the SARS-CoV-2 pandemic (trials start days between 4-2020 and first half of 2021).**

1. Thomas SJ, Moreira ED, Jr., Kitchin N, et al. Safety and Efficacy of the BNT162b2 mRNA Covid-19 Vaccine through 6 Months. N Engl J Med 2021; 385(19): 1761-73.
2. Ramasamy MN, Minassian AM, Ewer KJ, et al. Safety and immunogenicity of ChAdOx1 nCoV-19 vaccine administered in a prime-boost regimen in young and old adults (COV002): a single-blind, randomised, controlled, phase 2/3 trial. The Lancet 2020; 396(10267): 1979-93.
3. Baden LR, El Sahly HM, Essink B, et al. Efficacy and Safety of the mRNA-1273 SARS-CoV-2 Vaccine. N Engl J Med 2021; 384(5): 403-16.
4. Falsey AR, Sobieszczyk ME, Hirsch I, et al. Phase 3 Safety and Efficacy of AZD1222 (ChAdOx1 nCoV-19) Covid-19 Vaccine. N Engl J Med 2021; 385(25): 2348-60.
5. Sadoff J, Gray G, Vandebosch A, et al. Safety and Efficacy of Single-Dose Ad26.COV2.S Vaccine against Covid-19. N Engl J Med 2021; 384(23): 2187-201.
6. Tanriover MD, Doğanay HL, Akova M, et al. Efficacy and safety of an inactivated whole-virion SARS-CoV-2 vaccine (CoronaVac): interim results of a double-blind, randomised, placebo-controlled, phase 3 trial in Turkey. Lancet 2021; 398(10296): 213-22.
7. Halperin SA, Ye L, MacKinnon-Cameron D, et al. Final efficacy analysis, interim safety analysis, and immunogenicity of a single dose of recombinant novel coronavirus vaccine (adenovirus type 5 vector) in adults 18 years and older: an international, multicentre, randomised, double-blinded, placebo-controlled phase 3 trial. Lancet 2022; 399(10321): 237-48.
8. Hardt K, Vandebosch A, Sadoff J, et al. Efficacy, safety, and immunogenicity of a booster regimen of Ad26.COV2.S vaccine against COVID-19 (ENSEMBLE2): results of a randomised, double-blind, placebo-controlled, phase 3 trial. Lancet Infect Dis 2022; 22(12): 1703-15.
9. Hager KJ, Pérez Marc G, Gobeil P, et al. Efficacy and Safety of a Recombinant Plant-Based Adjuvanted Covid-19 Vaccine. N Engl J Med 2022; 386(22): 2084-96.
10. Toback S, Galiza E, Cosgrove C, et al. Safety, immunogenicity, and efficacy of a COVID-19 vaccine (NVX-CoV2373) co-administered with seasonal influenza vaccines: an exploratory substudy of a randomised, observer-blinded, placebo-controlled, phase 3 trial. Lancet Respir Med 2022; 10(2): 167-79.
11. Kremsner PG, Ahuad Guerrero RA, Arana-Arri E, et al. Efficacy and safety of the CVnCoV SARS-CoV-2 mRNA vaccine candidate in ten countries in Europe and Latin America (HERALD): a randomised, observer-blinded, placebo-controlled, phase 2b/3 trial. Lancet Infect Dis 2022; 22(3): 329-40.
12. Dai L, Gao L, Tao L, et al. Efficacy and Safety of the RBD-Dimer-Based Covid-19 Vaccine ZF2001 in Adults. N Engl J Med 2022; 386(22): 2097-111.
13. Khairullin B, Zakarya K, Orynbayev M, et al. Efficacy and safety of an inactivated whole-virion vaccine against COVID-19, QazCovid-in(R), in healthy adults: A multicentre, randomised, single-blind, placebo-controlled phase 3 clinical trial with a 6-month follow-up. EClinicalMedicine 2022; 50: 101526.
14. Dunkle LM, Kotloff KL, Gay CL, et al. Efficacy and Safety of NVX-CoV2373 in Adults in the United States and Mexico. N Engl J Med 2022; 386(6): 531-43.
15. Ali K, Berman G, Zhou H, et al. Evaluation of mRNA-1273 SARS-CoV-2 Vaccine in Adolescents. New England Journal of Medicine 2021; 385(24): 2241-51.
16. Walter EB, Talaat KR, Sabharwal C, et al. Evaluation of the BNT162b2 Covid-19 Vaccine in Children 5 to 11 Years of Age. N Engl J Med 2022; 386(1): 35-46.
17. Vadrevu KM, Reddy S, Jogdand H, et al. Immunogenicity and reactogenicity of an inactivated SARS-CoV-2 vaccine (BBV152) in children aged 2&#x2013;18 years: interim data from an open-label, non-randomised, age de-escalation phase 2/3 study. The Lancet Infectious Diseases 2022; 22(9): 1303-12.
18. Sridhar S, Joaquin A, Bonaparte MI, et al. Safety and immunogenicity of an AS03-adjuvanted SARS-CoV-2 recombinant protein vaccine (CoV2 preS dTM) in healthy adults: interim findings from a phase 2, randomised, dose-finding, multicentre study. Lancet Infect Dis 2022; 22(5): 636-48.
19. Bravo L, Smolenov I, Han HH, et al. Efficacy of the adjuvanted subunit protein COVID-19 vaccine, SCB-2019: a phase 2 and 3 multicentre, double-blind, randomised, placebo-controlled trial. Lancet 2022; 399(10323): 461-72.
20. Speich B, Chammartin F, Abela IA, et al. Antibody Response in Immunocompromised Patients After the Administration of Severe Acute Respiratory Syndrome Coronavirus 2 (SARS-CoV-2) Vaccine BNT162b2 or mRNA-1273: A Randomized Controlled Trial. Clin Infect Dis 2022; 75(1): e585-e93.
21. Lazarus R, Querton B, Corbic Ramljak I, et al. Immunogenicity and safety of an inactivated whole-virus COVID-19 vaccine (VLA2001) compared with the adenoviral vector vaccine ChAdOx1-S in adults in the UK (COV-COMPARE): interim analysis of a randomised, controlled, phase 3, immunobridging trial. Lancet Infect Dis 2022; 22(12): 1716-27.
22. Tabarsi P, Anjidani N, Shahpari R, et al. Evaluating the efficacy and safety of SpikoGen®, an Advax-CpG55.2-adjuvanted severe acute respiratory syndrome coronavirus 2 spike protein vaccine: a phase 3 randomized placebo-controlled trial. Clin Microbiol Infect 2023; 29(2): 215-20.
23. Chalkias S, Eder F, Essink B, et al. Safety, immunogenicity and antibody persistence of a bivalent Beta-containing booster vaccine against COVID-19: a phase 2/3 trial. Nat Med 2022; 28(11): 2388-97.
24. Moreira ED, Jr., Kitchin N, Xu X, et al. Safety and Efficacy of a Third Dose of BNT162b2 Covid-19 Vaccine. N Engl J Med 2022; 386(20): 1910-21.
25. Tabarsi P, Anjidani N, Shahpari R, et al. Immunogenicity and safety of SpikoGen®, an adjuvanted recombinant SARS-CoV-2 spike protein vaccine as a homologous and heterologous booster vaccination: A randomized placebo-controlled trial. Immunology 2022; 167(3): 340-53.
26. Hall VG, Ferreira VH, Ku T, et al. Randomized Trial of a Third Dose of mRNA-1273 Vaccine in Transplant Recipients. N Engl J Med 2021; 385(13): 1244-6.
27. Kumar D, Ferreira VH, Hall VG, et al. Neutralization of SARS-CoV-2 Variants in Transplant Recipients After Two and Three Doses of mRNA-1273 Vaccine : Secondary Analysis of a Randomized Trial. Ann Intern Med 2022; 175(2): 226-33.
28. Reindl-Schwaighofer R, Heinzel A, Mayrdorfer M, et al. Comparison of SARS-CoV-2 Antibody Response 4 Weeks After Homologous vs Heterologous Third Vaccine Dose in Kidney Transplant Recipients: A Randomized Clinical Trial. JAMA Intern Med 2022; 182(2): 165-71.
29. Temesgen Z, Burger CD, Baker J, et al. Lenzilumab in hospitalised patients with COVID-19 pneumonia (LIVE-AIR): a phase 3, randomised, placebo-controlled trial. The Lancet Respiratory Medicine 2022; 10(3): 237-46.
30. Tixagevimab-cilgavimab for treatment of patients hospitalised with COVID-19: a randomised, double-blind, phase 3 trial. Lancet Respir Med 2022; 10(10): 972-84.
31. Gupta A, Gonzalez-Rojas Y, Juarez E, et al. Early Treatment for Covid-19 with SARS-CoV-2 Neutralizing Antibody Sotrovimab. N Engl J Med 2021; 385(21): 1941-50.
32. Streinu-Cercel A, Săndulescu O, Preotescu LL, et al. Efficacy and Safety of Regdanvimab (CT-P59): A Phase 2/3 Randomized, Double-Blind, Placebo-Controlled Trial in Outpatients With Mild-to-Moderate Coronavirus Disease 2019. Open Forum Infect Dis 2022; 9(4): ofac053.
33. Hyperimmune immunoglobulin for hospitalised patients with COVID-19 (ITAC): a double-blind, placebo-controlled, phase 3, randomised trial. Lancet 2022; 399(10324): 530-40.
34. Cohen MS, Nirula A, Mulligan MJ, et al. Effect of Bamlanivimab vs Placebo on Incidence of COVID-19 Among Residents and Staff of Skilled Nursing and Assisted Living Facilities: A Randomized Clinical Trial. Jama 2021; 326(1): 46-55.
35. Weinreich DM, Sivapalasingam S, Norton T, et al. REGEN-COV Antibody Combination and Outcomes in Outpatients with Covid-19. N Engl J Med 2021; 385(23): e81.
36. Levin MJ, Ustianowski A, De Wit S, et al. Intramuscular AZD7442 (Tixagevimab–Cilgavimab) for Prevention of Covid-19. New England Journal of Medicine 2022; 386(23): 2188-200.
37. Dougan M, Nirula A, Azizad M, et al. Bamlanivimab plus Etesevimab in Mild or Moderate Covid-19. N Engl J Med 2021; 385(15): 1382-92.
38. Casirivimab and imdevimab in patients admitted to hospital with COVID-19 (RECOVERY): a randomised, controlled, open-label, platform trial. Lancet 2022; 399(10325): 665-76.
